# Postmortem Stability of SARS-CoV-2 in Nasopharyngeal Mucosa

**DOI:** 10.3201/eid2701.203112

**Published:** 2021-01

**Authors:** Fabian Heinrich, Kira Meißner, Felicia Langenwalder, Klaus Püschel, Dominik Nörz, Armin Hoffmann, Marc Lütgehetmann, Martin Aepfelbacher, Eric Bibiza-Freiwald, Susanne Pfefferle, Axel Heinemann

**Affiliations:** University Medical Center Hamburg-Eppendorf, Hamburg, Germany

**Keywords:** SARS-CoV-2, COVID-19, respiratory infections, severe acute respiratory syndrome coronavirus 2, 2019 novel coronavirus disease, coronavirus disease, zoonoses, viruses, coronavirus, postmortem, RNA stability, infectivity

## Abstract

Analyses of infection chains have demonstrated that severe acute respiratory syndrome coronavirus 2 is highly transmissive. However, data on postmortem stability and infectivity are lacking. Our finding of nasopharyngeal viral RNA stability in 79 corpses showed no time-dependent decrease. Maintained infectivity is supported by virus isolation up to 35 hours postmortem.

Detailed analyses of severe acute respiratory syndrome coronavirus 2 (SARS-CoV-2) transmission have shown the virus to be highly transmissible through droplet and contact-transmitted viral spreading; reproduction indices were 2.2–3.6 (*1*). Amid the coronavirus disease (COVID-19) pandemic, case-fatality rates of up to 9.26% occur in areas hard-struck by SARS-CoV-2 (*2*). The likelihood of virus transmission through deceased persons remains unclear. However, in recent pandemics of influenza, high and sustainable virus stability and infectivity within corpses were demonstrated (*3*,*4*), necessitating careful and conscious handling. To determine the possibility of SARS-CoV-2 transmission through deceased persons, we conducted a study of postmortem viral RNA stability.

The federal state of Hamburg, Germany, has mandated autopsies since March 2020 in accordance with the German Infection Protection Act for all patients with reverse transcription PCR (RT-PCR)–confirmed SARS-CoV-2 infection. Data and sample acquisition for the study were performed during March 22–May 1, 2020. To confirm the initial diagnosis and quantify the viral load in the corpses, nasopharyngeal swab samples (ESwab; Copan, https://products.copangroup.com) were taken at patient admission to the Department of Legal Medicine (University Medical Center Hamburg-Eppendorf). Corpses were stored at 4°C in the refrigerator. Antemortem and postmortem nasopharyngeal swab samples were taken according to recent standards (*5*) by trained, medically qualified personnel to ensure maximum reliability and consistent quality. Samples were analyzed for SARS-CoV-2 RNA as described previously (*6*).

The Ethics Committee of the Hamburg Chamber of Physicians approved the study (no. PV7311). The local clinical institutional review board, complying with the Declaration of Helsinki, also approved the study.

Antemortem nasopharyngeal swab samples (Appendix Figure) were collected by medical staff at the intensive care unit of the University Medical Center Hamburg and by general practitioners from on-call duty at a median of 6 days (range 2–14 [interquartile range (IQR) 6.3]) before death (n = 10). Using a Wilcoxon test for paired data, we did not detect any effect of the event of death on the SARS-CoV-2 RNA load (U= −5; p = 0.85). We found no correlation between the postmortem interval (time of death until cooling at 4°C; median 17.8 [range 2.7–482.6]) hours and the viral RNA loads of corpses, as indicated by Spearman correlation of 79 matched datasets (Figure, panel A).

**Figure:**
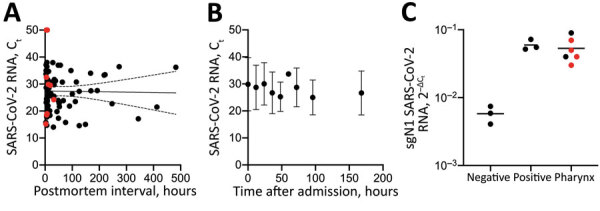
Postmortem stability of SARS-CoV-2 in nasopharyngeal mucosa. A) Correlation of SARS-CoV-2 RNA loads of the pharynx (at corpse admission to the Department of Legal Medicine) with the postmortem interval (time of death until cooling at 4°C) in 79 matched datasets. Red indicates patients in the longitudinal cohort. Spearman R = –0.07; 2-tailed p = 0.5. B) Median SARS-CoV-2 RNA loads with 95% CIs (error bars) in a series of 9 sequential pharyngeal swab samples (time points 0, 12, 24, 36, 48, 60, 72, 96, and 168 hours after admission) for 11 corpses. C) sgN1 RNA loads of SARS-CoV-2 in pharyngeal tissue of 6 corpses. Negative and positive controls from SARS-CoV-2 cell cultures. Red indicates samples with successful virus isolation from pharyngeal tissue (S. Pfefferle, unpub. data, https://doi.org/10.1101/2020.10.10.334458). Negative results are reflected by C_t_ 50. Ct, cycle threshold; SARS-CoV-2, severe acute respiratory syndrome coronavirus 2; sgN1 RNA, subgenomic RNA loads of the N1-gene.

To analyze postmortem stability of SARS-CoV-2 RNA, we selected 11 corpses with short postmortem intervals for a detailed observation over 7 days (168 hours) (Table). The median postmortem interval was 5.7 (range 2.9–32.0 [IQR 6.9]) hours. The median cycle threshold (C_t_) of SARS-CoV-2 RNA in swab samples taken at admission was 29.52 (range 15.2–50.0 [IQR 22.5]) (Figure, panel A). We determined viral load in a series of 9 sequential pharyngeal swab samples (time points 0, 12, 24, 36, 48, 60, 72, 96, and 168 hours after admission). We consistently detected SARS-CoV-2 RNA at constant levels at all time points analyzed (Figure, panel B), except for patient 7 at 0, 12, and 24 hours after admission and patient 8 at admission. Because subsequent samples were positive for all corpses, we attributed those discrepancies to deviations in the sample collection. A general mixed model found no time-dependent effect on SARS-CoV-2 RNA loads (estimate −0.06, SE 0.01; p = 0.58) (*7*). Because of impaired interval-scaling of metric variables, we excluded negative C_t_ values from the statistical analysis. Intriguingly, the estimate suggests an increase of the viral load without revealing significant results (0.6%/hour).

**Table:** Basic clinical information about and autopsy findings of patients in a longitudinal follow-up cohort and for virus isolation, Department of Legal Medicine, Hamburg, Germany, 2020*

Patient no.	Age, y/sex	BMI, kg/m^2^	Main autopsy finding	Disease duration, d	Postmortem interval, h†	Postmortem SARS-CoV-2 RNA load at admission, C_t_‡
1§	54/F	29.6	Pneumonia	5	11.92	29.86
2§	66/M	25.3	Pneumonia	ND	32.03	24.22
3§	63/M	37.3	Pulmonary embolism, pneumonia	6	5.03	32.55
4§	70/M	22.2	Pneumonia, bronchitis, respiratory failure	6	7.48	18.97
5§	52/M	38.8	Pulmonary embolism	10	5.32	ND
6§	90/F	24.9	Pneumonia, aspiration	13	19.35	29.52
7§	71/M	ND	Pneumonia, MODS	ND	7.87	50
8§	77/M	33.2	Pneumonia	18	5.08	50
9§	61/M	32.3	Intracerebral hemorrhage, pneumonia	ND	4.37	ND
10§	76/M	37.7	Pneumonia, MODS, endocarditis, leukemia	ND	2.85	15.22
11§,¶	59/F	22.2	Pneumonia, multiple myeloma	18	5.67	18.55
12¶	83/F	26.0	Pneumonia, non-Hodgkin lymphoma	25	6.83	ND
13¶	80/M	28.5	Pulmonary embolism, pneumonia, myelofibrosis	12	6.5	ND
14¶	71/F	29.0	Pneumonia, myelofibrosis	25	12.1	ND
15¶	84/F	21.4	Pneumonia	ND	35.75	ND
16¶	31/M	20.6	Pneumonia, germ cell tumor	ND	9.08	ND

*Ct, cycle threshold; MODS, multiple organ dysfunction syndrome; ND, not determined; SARS-CoV-2, severe acute respiratory syndrome coronavirus 2.

†Time of death until cooling at 4°C.

‡Negative results are reflected by Ct 50.

§Longitudinal cohort.

¶Virus isolation cohort (S. Pfefferle, unpub. data, https://doi.org/10.1101/2020.10.10.334458).

Six patients in this study (patients 11–16) previously were part of a study in which virus growth from different tissues (including pharynx) of patients dying of RT-PCR–confirmed SARS-CoV-2 infection was investigated (S. Pfefferle, unpub. data, https://doi.org/10.1101/2020.10.10.334458) (Table). That study showed that replicating virus was detected in the throat of patients up to 35.8 hours after death. Both the detection of subgenomic RNA (sgRNA) by next-generation sequencing and virus growth could be shown in those throat samples. We also detected sgRNA by RT-PCR in throat tissue samples of these 6 previously published patients (*8**–**10*) (Figure, panel C); samples in which virus could be cultivated (S. Pfefferle, unpub. data, https://doi.org/10.1101/2020.10.10.334458) are highlighted in red.

We demonstrated maintained infectivity of SARS-CoV-2 in tissues of deceased patients. SARS-CoV-2 RNA persisted over time at constantly high titers. Taken together, our data indicate potentially high infectivity of human corpses, requiring hazard assessments in professional fields concerned and careful and conscious handling.

Our infectivity study relies on a limited number of cases and patients with severe immunosuppression. Further research should investigate viral persistence in corpses with longer postmortem intervals (>1 week) and corpses exhibiting lower initial viral loads. We recommend all work on corpses be conducted according to guidelines recently published by the World Health Organization, especially in the framework of widespread death in pandemics (https://apps.who.int/iris/rest/bitstreams/1300088/retrieve).

Appendix SARS-CoV-2 RNA loads from matched antemortem and postmortem nasopharyngeal swab samples.
