# Supplementary material for: Postmortem Stability of SARS-CoV-2 in Nasopharyngeal Mucosa
Source: Emerg Infect Dis. 2021 Jan;27(1):329–31. doi: 10.3201/eid2701.203112 (PMC7774572; doi:10.3201/eid2701.203112)
Supplement: Appendix — SARS-CoV-2 RNA loads from matched antemortem and postmortem nasopharyngeal swab samples. [file 20-3112-Techapp-s1.pdf]

Article DOI: https://doi.org/10.3201/eid2701.203112

Postmortem Stability of SARS-CoV-2 in Nasopharyngeal Mucosa

Appendix

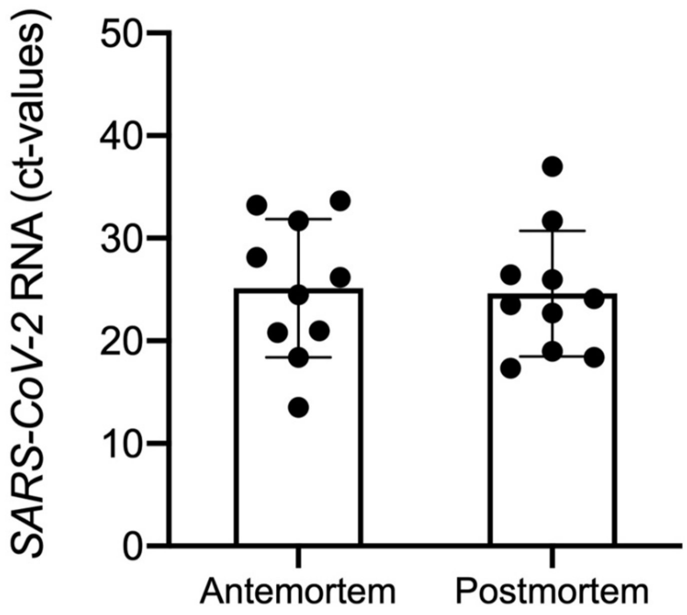

Appendix Figure. SARS-CoV-2 RNA loads (cycle threshold values) from matched antemortem and postmortem nasopharyngeal swab samples, illustrating 10 corpses from Figure 1, panel A, in the main text.
